# Activation of Wnt/β-Catenin Signaling Increases Apoptosis in Melanoma Cells Treated with Trail

**DOI:** 10.1371/journal.pone.0069593

**Published:** 2013-07-15

**Authors:** Zachary F. Zimmerman, Rima M. Kulikauskas, Karol Bomsztyk, Randall T. Moon, Andy J. Chien

**Affiliations:** 1 Department of Medicine, the University of Washington School of Medicine, Seattle, Washington, United States of America; 2 The Howard Hughes Medical Institute, Chevy Chase, Maryland, United States of America; The Moffitt Cancer Center & Research Institute, United States of America

## Abstract

While the TRAIL pathway represents a promising therapeutic target in melanoma, resistance to TRAIL-mediated apoptosis remains a barrier to its successful adoption. Since the Wnt/β-catenin pathway has been implicated in facilitating melanoma cell apoptosis, we investigated the effect of Wnt/β-catenin signaling on regulating the responses of melanoma cells to TRAIL. Co-treatment of melanoma cell lines with WNT3A-conditioned media and recombinant TRAIL significantly enhanced apoptosis compared to treatment with TRAIL alone. This apoptosis correlates with increased abundance of the pro-apoptotic proteins BCL2L11 and BBC3, and with decreased abundance of the anti-apoptotic regulator Mcl1. We then confirmed the involvement of the Wnt/β-catenin signaling pathway by demonstrating that siRNA-mediated knockdown of an intracellular β-catenin antagonist, AXIN1, or treating cells with an inhibitor of GSK-3 also enhanced melanoma cell sensitivity to TRAIL. These studies describe a novel regulation of TRAIL sensitivity in melanoma by Wnt/β-catenin signaling, and suggest that strategies to enhance Wnt/β-catenin signaling in combination with TRAIL agonists warrant further investigation.

## Introduction

The incidence of melanoma has increased in recent decades, and it has become a major cause of cancer related morbidity and mortality. Despite a growing understanding of the molecular pathogenesis of melanoma and the development of promising new therapies, overall survival with advanced/ metastatic disease remains poor [1]. Due to the limited efficacy of standard chemotherapies, intensive research has focused on the development of alternative approaches to promote melanoma cell death. TNF Receptor Death-Inducing Ligand (encoded by *TRAIL*; also called Apo2L) is a member of the TNF superfamily of cytokines. TRAIL can potently induce apoptosis upon ligation of its cognate receptors Death Receptors 4 and 5 (encoded by *DR4* and *DR5*, respectively) in many tumors including melanoma [2,3]. TRAIL characteristically spares normal tissue, thus making it an attractive target for cancer drug development. Additionally, TRAIL also appears to be involved in the immune response against melanoma [2,4]. Despite its therapeutic promise, melanoma cell lines and freshly isolated tumors are often resistant to TRAIL-mediated apoptosis, thereby limiting the potential efficacy of TRAIL agonists [5].

TRAIL binding triggers the extrinsic apoptotic pathway through recruitment of the adapter protein FADD and the subsequent formation of the death induced signaling complex (DISC). Procaspase-8 is then recruited to the DISC through an interaction with its death effector domain (DED) which results in its autocatalytic activation and subsequent initiation of downstream apoptotic effector proteins. Caspase-8 can also trigger the mitochondrial apoptotic cascade through cleavage of BID, a pro-apoptotic member of the Bcl-2 family of proteins. In melanoma, regulation of TRAIL sensitivity can occur both at the level of the extrinsic (death receptor) and intrinsic (mitochondrial) apoptotic pathways (reviewed in 6).

The Wnt/β-catenin signaling pathway plays a prominent role in development, cell fate determination, and cancer [7]. In the absence of Wnt ligands, the serine/threonine kinase Glycogen Synthase Kinase-3 (encoded by *GSK-3*) phosphorylates β-catenin, targeting it to the proteasome for degradation. Wnt ligands including WNT3A bind to the Frizzled and LRP5/6 families of co-receptors. This binding promotes the stabilization of β-catenin (encoded by *CTNNB1*) in the cytosol followed by its eventual nuclear translocation to effect changes in target gene transcription [8]. Evidence from melanocyte development, genetic mouse models, and human patients implicate Wnt/β-catenin signaling in melanoma pathogenesis [9–16]. Unlike most cancers, where Wnt signaling is considered to be a driver of oncogenesis, the precise role of Wnt/ β-catenin signaling in melanoma remains unclear (reviewed in 11). For example, whereas stabilized constitutively active β-catenin seems to promote melanomagenesis and metastasis in mice, high cytoplasmic and nuclear β-catenin levels (a surrogate indicator of pathway activity) in patient derived tumors correlates with a more favorable prognosis [9,10,12,17]. Furthermore, WNT3A overexpression in mouse or human melanoma cell lines inhibits *in vitro* proliferation and *in vivo* tumor growth [9,18]. Together these studies suggest that Wnt/β-catenin signaling may have differing effects in melanoma depending on the context in which the signal is received.

Recently, we found that activation of Wnt/β-catenin signaling can enhance apoptosis in melanoma cells treated with targeted BRAF inhibitors. Given the potential promise of TRAIL-based therapy and the problem of intrinsic resistance to TRAIL in melanoma, we sought to address the question of whether Wnt/β-catenin signaling could also sensitize melanoma cells to TRAIL-induced apoptosis. Our study demonstrates that activation of the Wnt/β-catenin pathway in melanoma significantly enhances apoptosis triggered by recombinant human TRAIL (rhTRAIL). Moreover, elevating β-catenin signaling with the small molecule GSK-3 inhibitor CHIR99021 or by siRNA-mediated knockdown of a negative regulator of β-catenin, AXIN1, efficiently sensitized melanoma cells to rhTRAIL. Conversely, β-catenin siRNAs blocked apoptosis. These results have implications for the development of improved strategies to target the TRAIL pathway for melanoma therapy.

## Results

### Wnt/β-catenin signaling sensitizes melanoma cell lines to TRAIL-mediated apoptosis

**Figure 1:**
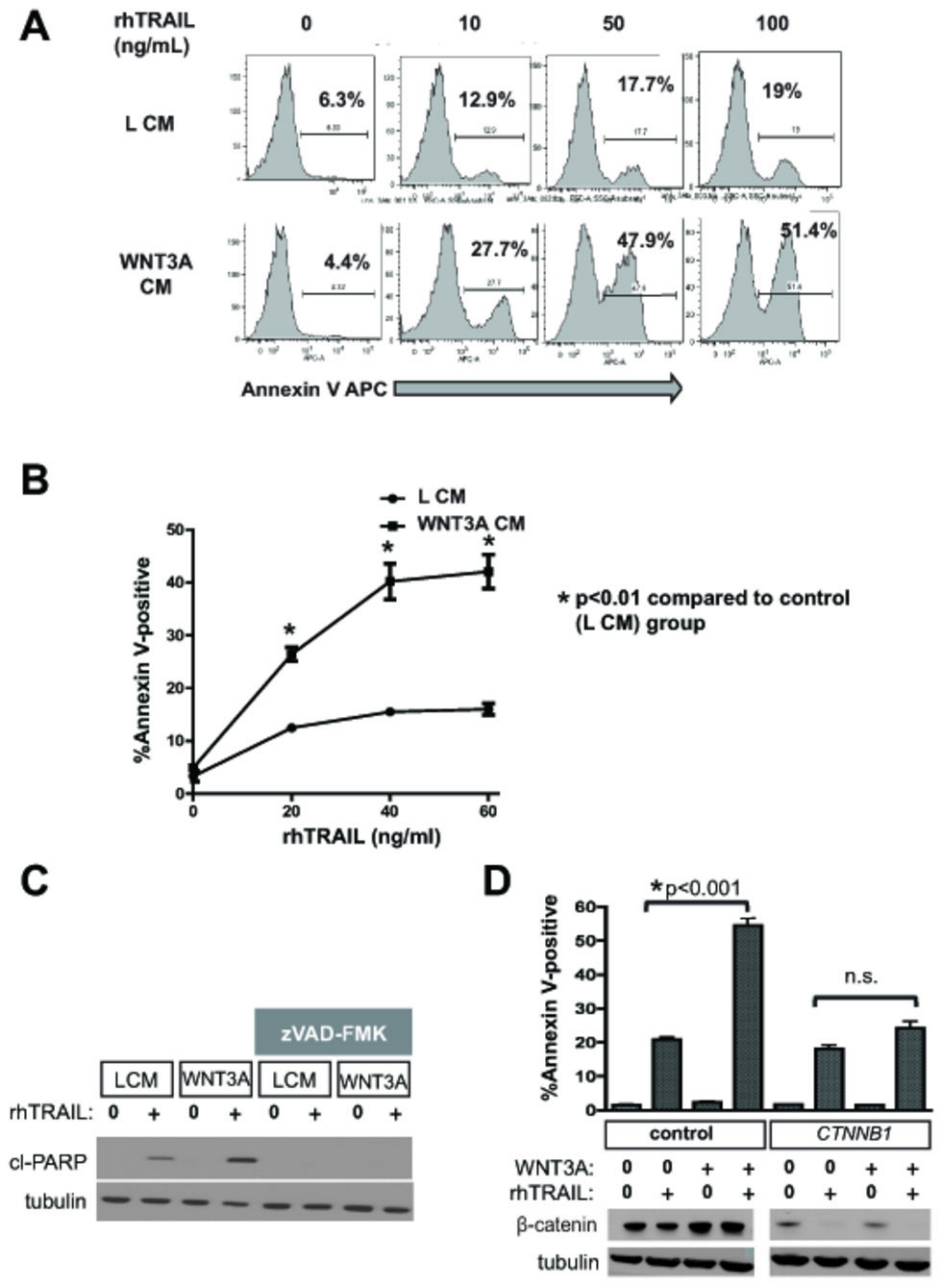
WNT3A sensitizes melanoma cell lines to TRAIL in a β-catenin-dependent manner. A) A375 melanoma cells were treated with indicated doses of rhTRAIL for 24 hours in the presence of WNT3A or control L-cell (L) CM (10%). Apoptotic cells were detected by Annexin V binding assay using FACS. Representative FACS histograms with Annexin V-positive gates are shown. Percent apoptotic cells are indicated. B) A375 cells were treated with indicated doses of rhTRAIL in the presence of WNT3A or L CM (10%). Data represents mean % apoptotic cells ± SEM as determined by Annexin V positivity at 24 hours. An (*) indicates that the difference between L and WNT3A CM treated cells at the indicated TRAIL dose is significant with a p-value of <0.01, calculated using Student’s t-test. C) A375 cells were treated with rhTRAIL and WNT3A in the absence and presence of the pan-caspase inhibitor zVAD-FMK (100 µM), and then analyzed for cleaved PARP at 24 hours. C) A375 cells were pre-treated with siRNA specific for β-catenin (*CTNNB1*) or non-targeting control siRNA for 48 hours. Cells were then treated with rhTRAIL (20 ng/mL) in the presence of WNT3A CM or L CM. Data represents mean percentages of Annexin V-positive cells (+/- SEM) at 24 hours post-treatment as detected by FACS. P-values were calculated using one way ANOVA and Tukey’s post-test analysis. A parallel immunoblot (lower panel) confirms knockdown of β-catenin. The experiments in A and B are representative of at least three independent experiments with similar results.

We first asked whether Wnt/β-catenin signaling could enhance TRAIL-mediated apoptosis in melanoma. Apoptosis, as assessed by Annexin V-positive cells, was observed in A375 human melanoma cells treated with rhTRAIL in a dose-dependent fashion (Figure 1A,B) as has been previously described [2]. Consistent with our hypothesis, concurrent treatment with WNT3A conditioned media (CM) markedly enhanced the percentage of cells undergoing apoptosis at all doses of rhTRAIL tested (Figure 1A,B). The observed apoptosis was higher in WNT3A conditioned media at all concentrations tested and maximal at 24 hours post-treatment (Supplemental Figure S1A, B). An alternate marker of apoptosis, cleaved PARP, was also measurably elevated when A375 cells were treated with WNT3A CM in the presence of rhTRAIL compared with controls (Figure 1C). As further confirmation of caspase-mediated apoptosis, cleavage of PARP with TRAIL and TRAIL plus WNT3A was inhibited upon treatment with the pan-caspase inhibitor zVAD-FMK (Figure 1C).

The downstream effector of WNT3A is β-catenin (encoded by *CTNNB1*). To determine if β-catenin is required for WNT3A-mediated sensitization of melanoma to rhTRAIL, A375 cells were pre-treated with β-catenin-specific siRNA prior to treatment with WNT3A CM and rhTRAIL. Knockdown of β-catenin completely abrogated the effect of WNT3A on apoptosis, indicating that β-catenin-dependent signaling is required for sensitization to rhTRAIL (Figure 1D).

**Figure 2:**
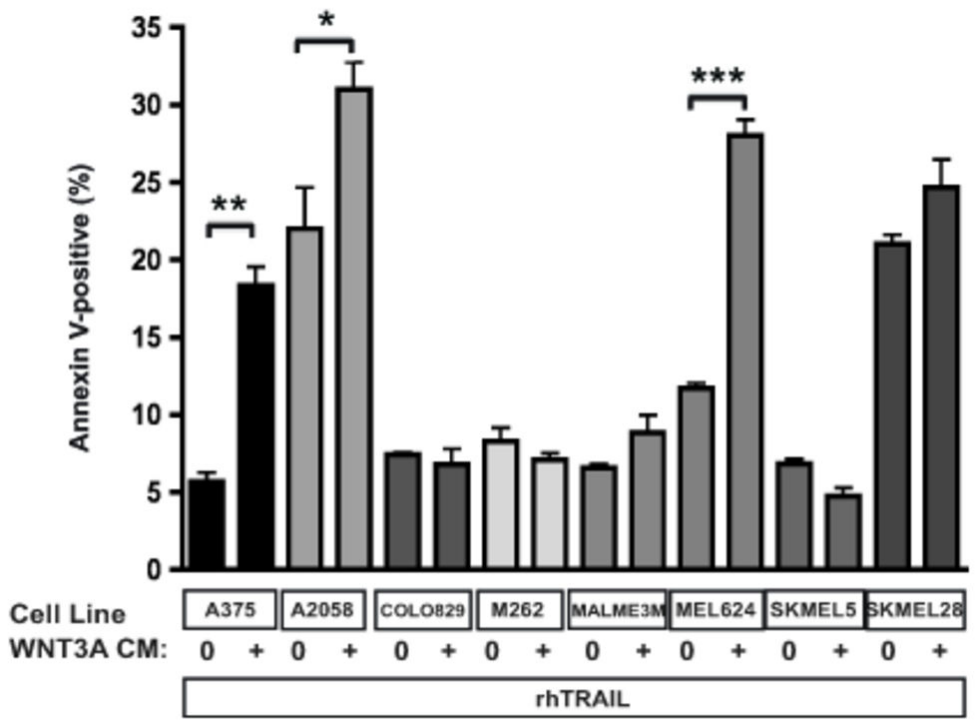
Melanoma cells exhibit diverse apoptotic response to WNT3A plus TRAIL. Several human melanoma cell lines were assessed for apoptosis at 24 hours upon treatment with rhTRAIL (20 ng/ml) in the absence (10% L CM) and presence of 10% WNT3A CM. Statistically significant increases in Annexin V-positive cells were seen in three cell lines. Data represents the mean percentage of apoptotic cells for three replicates/group (* p<0.05; **p<0.01; ***p<0.001).

Substantial heterogeneity has been described in the sensitivity to TRAIL of human melanoma cell lines and patient-derived primary tumors [5]. Heterogeneity has also been described in the ability of WNT3A to enhance apoptosis triggered by the inhibition of oncogenic BRAF [18]. We therefore tested a panel of melanoma cell lines for their sensitivity to rhTRAIL in the presence of WNT3A. WNT3A treatment significantly enhanced TRAIL-dependent apoptosis in multiple melanoma cell lines, but did so in a heterogeneous fashion with some lines being unaffected by WNT3A in terms of TRAIL sensitivity (Figure 2).

### Wnt/β-catenin signaling increases levels of pro-apoptotic BCL2L11 and BBC3 and diminishes levels of the anti-apoptotic protein MCL1

**Figure 3:**
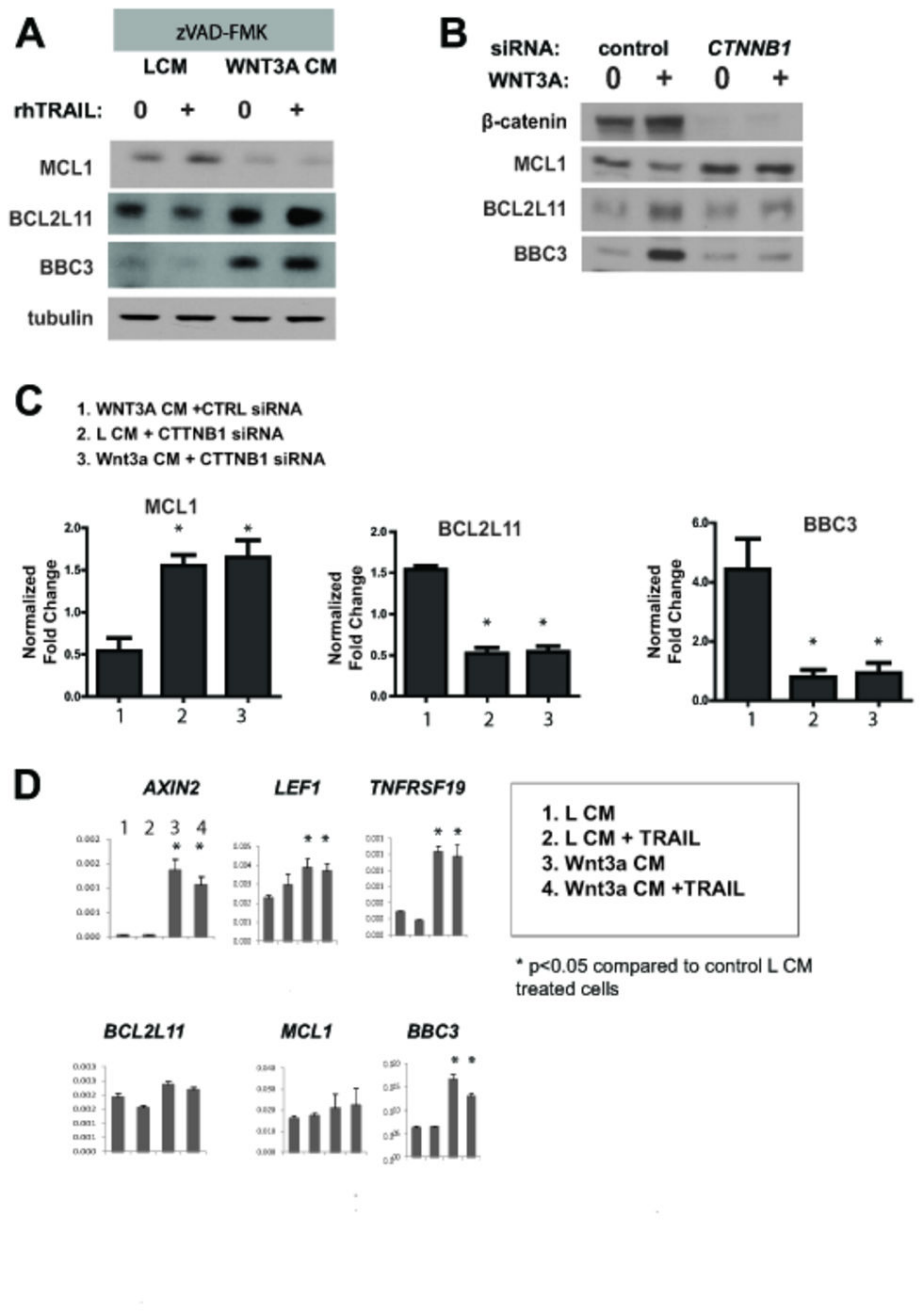
Wnt/β-catenin activation enhances the expression of the pro-apoptotic proteins BIM and PUMA and diminishes levels of anti-apoptotic MCL1. A) A375 melanoma cells were treated with rhTRAIL (20 ng/mL) in the presence of WNT3A or L CM. To inhibit caspase activity, cells were also treated with 100µM zVAD-FMK. Immunoblotting analysis was performed with the indicated antibodies. B) A375 cells were pre-treated with β-catenin (*CTNNB1*) versus control siRNA. 48 hours later, the cells were treated with WNT3A CM or L CM. Immunoblot analysis was then performed with the indicated antibodies. C) Graphs represent the fold-change in protein expression in (B) from digitally-quantified immunoblots +/- SEM (n=5 blots from independent experiments). Each condition was normalized to control cells treated with L CM + control siRNA. D) A375 melanoma cells were treated with WNT3A CM versus L CM in the presence or absence of rhTRAIL (20ng/mL) in the presence of zVAD-FMK (100µM). After 24 hours, RNA was collected for cDNA synthesis and analysis by quantitative real-time PCR (qRT-PCR). Data represents mean relative expression of the indicated gene based on three replicates/group. Significant differences between groups are indicated.

Sensitivity of melanoma to TRAIL can be regulated at the level of the extrinsic death receptor pathway as well as the intrinsic, mitochondrial dependent pathway [6]. We therefore examined levels of several known or predicted regulators of TRAIL dependent-apoptosis in melanoma cells treated with WNT3A CM in the presence of the pan-caspase inhibitor zVAD-FMK. Notably, abundance of the pro-apoptotic BH3-domain only proteins BIM (encoded by *BCL2L11*) and PUMA (encoded by *BBC3*) was measurably increased in the presence of WNT3A. Additionally, abundance of the anti-apoptotic Bcl-2 family member MCL1 was significantly diminished upon WNT3A treatment (Figure 3A). Supporting the requirement of β-catenin for the enhancement of TRAIL-dependent apoptosis, siRNA mediated knockdown of β-catenin rescued the changes in BCL2L11, BBC3, and MCL1 protein abundance observed with WNT3A treatment (Figure 3B and 3C).

Since BCL2L11 and BBC3 can be regulated both at the transcriptional and post-transcriptional level, we examined transcription of these genes by quantitative real-time PCR (qPCR) [19,20]. In these experiments, cells were again treated with zVAD-FMK to exclude the possibility that any transcriptional changes observed were masked due to apoptosis. AXIN2, like AXIN1, is a negative regulator of β-catenin signaling; however, unlike AXIN1 it is transcriptionally regulated by Wnt/β-catenin signaling and functions as a negative feedback regulator of the pathway [21]. Significant transcriptional up-regulation of *AXIN2* along with other WNT3A target genes, *LEF1* and *TNFRSF19*, was observed as expected. *BCL2L11* and *MCL1* mRNA levels were unaffected by WNT3A treatment, suggesting a post-transcriptional mechanism is involved in Wnt-dependent regulation of BCL2L11 and MCL1 abundance (Figure 3A). In contrast, *BBC3* transcript was significantly up-regulated by WNT3A treatment (Figure 3D). In addition, we failed to observe transcriptional changes in a panel of other apoptosis-related genes in WNT3A treated cells (Supplemental Figure S2).

### Small Molecule Activation of Wnt/β-catenin Signaling Enhances TRAIL-Dependent Apoptosis in Melanoma

**Figure 4:**
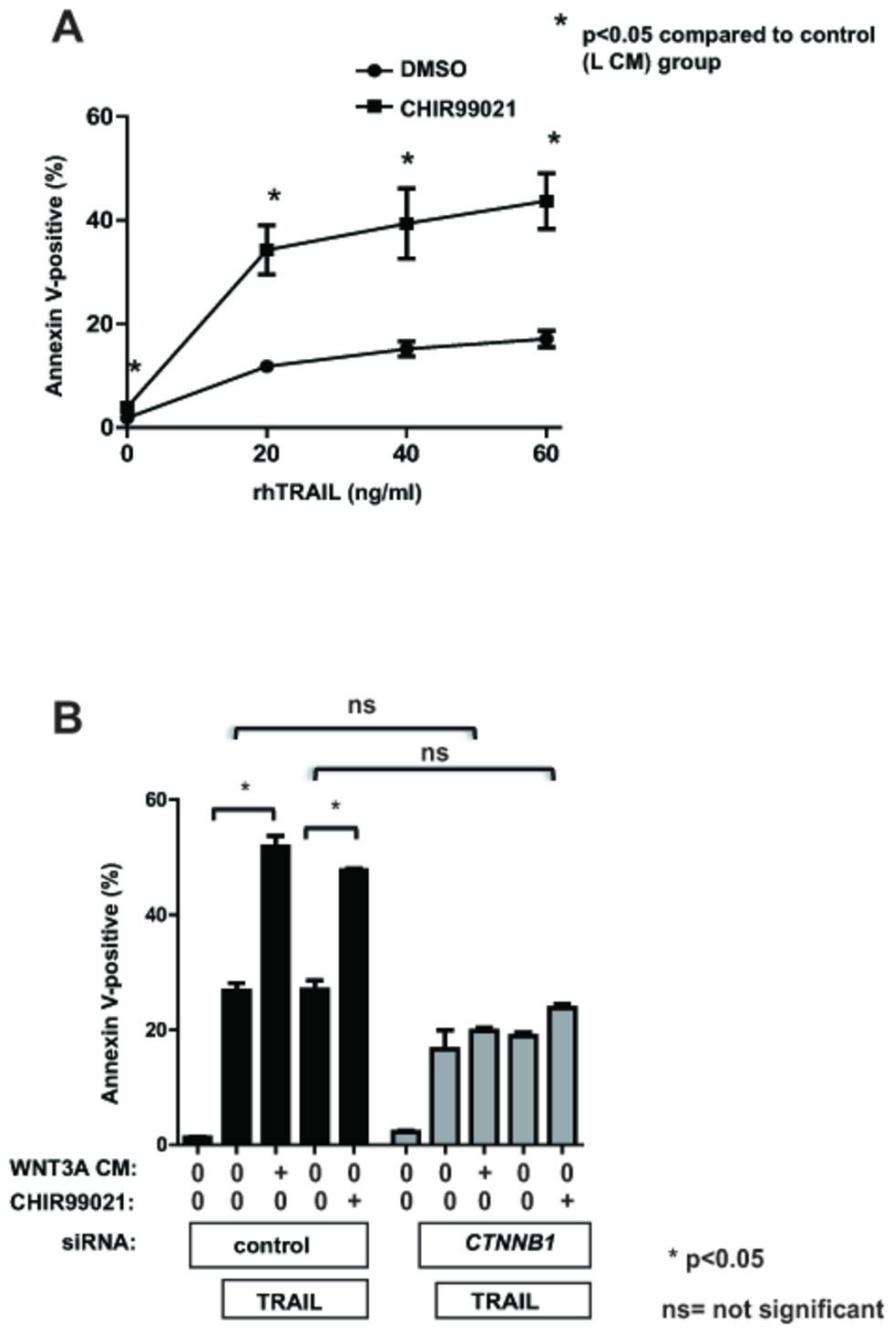
Inhibition of GSK-3 sensitizes melanoma cells to TRAIL-mediated apoptosis. A) A375 melanoma cells were treated with rhTRAIL at doses indicated on x-axis. Cells were concurrently treated with the GSK-3 inhibitor CHIR99021 (5µM) or DMSO vehicle control (v/v). Data represents the percentage of apoptotic cells measured by Annexin V-positive cells at 24 hours post-treatment by FACS. B) A375 cells were pre-treated with control siRNA or siRNA specific for β-catenin. 48 hours later, cells were treated with rhTRAIL (20ng/mL). 24 hours post-treatment, the percentage of apoptotic cells was determined by AnnexinV binding using FACS. Data represents mean percentage of apoptotic cells (+/- SEM). P-values were calculated by one-way ANOVA with a Tukey’s post-test analysis.

Activation of Wnt/β-catenin signaling triggers a series of molecular events that involve inhibition of the kinase GSK-3 (encoded by *GSK-3*), thereby inhibiting the phosphorylation of β-catenin, inhibiting proteasomal degradation, and enhancing accumulation of β-catenin in the cytosol and nucleus [22]. As a result, GSK-3 inhibitors have been used experimentally as pharmacologic activators of Wnt/β-catenin signaling. We examined the effect of an established GSK-3 inhibitor, CHIR99021, on TRAIL-dependent apoptosis [23]. A375 cells treated with CHIR99021 exhibited a dose-dependent increase in the percentage of cells undergoing apoptosis upon rhTRAIL treatment compared to control treated cells (Figure 4A). Enhancement of apoptosis by CHIR99021 was partially dependent on β-catenin, since siRNA-mediated knockdown of β-catenin (encoded by *CTNNB1*) partially inhibited the enhanced apoptosis seen with CHIR99021 treatment (Figure 4B).

### AXIN1 inhibits TRAIL-dependent apoptosis in melanoma

**Figure 5:**
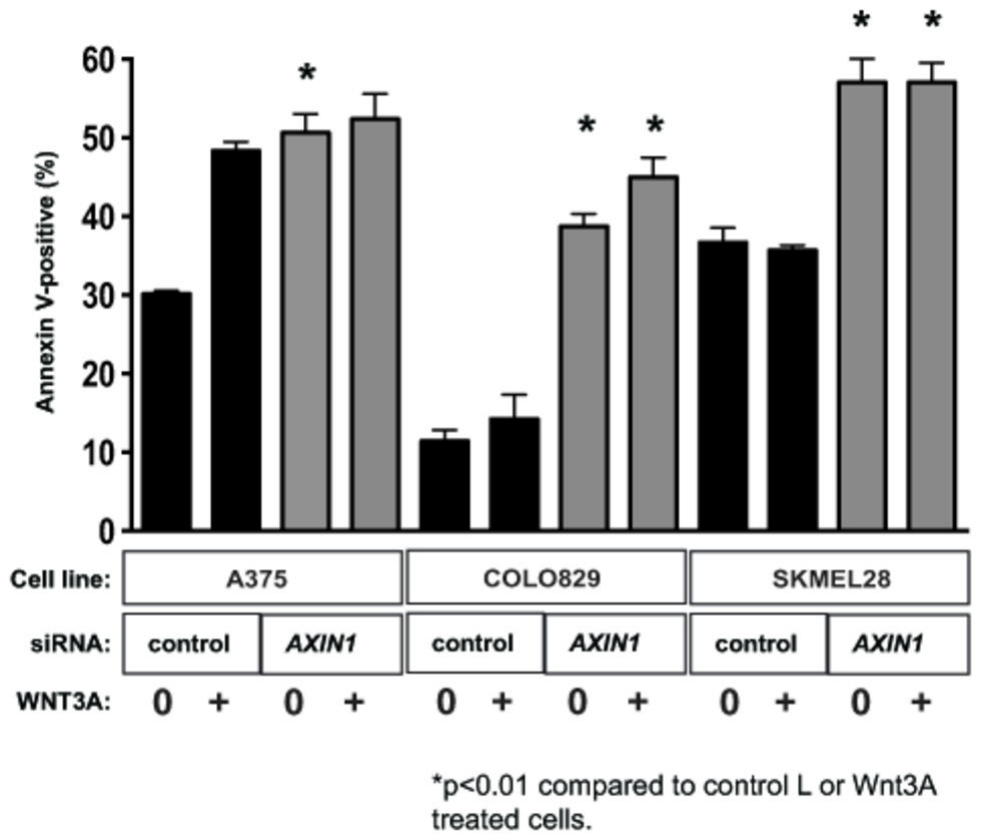
Depletion of AXIN1 sensitizes melanoma cells to TRAIL-mediated apoptosis. Melanoma cell lines indicated were pre-treated with control versus *AXIN1*-specific siRNA for 48 hours. Cells were then treated either WNT3A CM or L CM in addition to rhTRAIL (20ng/mL). The percentage of apoptotic cells was determined by Annexin V binding using FACS. Data represents the mean percentage of apoptotic cells at 24 hours post-treatment +/- SEM. P-values were calculated by one-way ANOVA with a Tukey’s post-test analysis. * indicates p <0.05 compared to either L or WNT3A cells treated with control siRNA.

AXIN1 is a scaffold protein that serves as a key negative regulator of Wnt/β-catenin signaling [24]. Previously we have shown that AXIN1 levels are regulated by the ERK/MAPK signaling pathway in melanoma, and that depletion of AXIN1 by siRNA-mediated knockdown significantly sensitized multiple melanoma cell lines to apoptosis when treated with a targeted small molecule inhibitor of oncogenic BRAF [18]. To determine the effect of AXIN1 depletion on TRAIL-mediated apoptosis, we pre-treated melanoma cell lines with siRNA specific for AXIN1 prior to treatment with TRAIL and WNT3A CM. We targeted two cell lines, COLO829 and SKMEL28, which exhibited no significant apoptosis with TRAIL plus WNT3A (Figure 2). AXIN1-depleted cells were significantly more sensitive to TRAIL compared with control siRNA-treated cells across three distinct human melanoma cell lines (Figure 5). These results suggest that AXIN1 is a regulator of sensitivity to TRAIL-induced apoptosis in melanoma, and that decreasing AXIN1 levels can confer sensitivity to apoptosis with TRAIL in previously insensitive cell lines.

## Discussion

Targeting the TRAIL pathway is a potentially promising strategy for the treatment of advanced melanoma since TRAIL can selectively kill cancer cells while sparing non-cancerous tissue [3]. Melanoma cells are characteristically resistant to apoptosis, and the observed variability in sensitivity to TRAIL has been a primary roadblock to the clinical application of TRAIL agonists [5]. These results suggest that pathways such as Wnt/β-catenin signaling can potentially be leveraged to modulate cellular responses to TRAIL agonists.

In this study we have demonstrated that the GSK-3 inhibitor CHIR99021 sensitizes melanoma cells to TRAIL-mediated apoptosis. Notably, inhibition of GSK-3 has been previously observed to enhance cell death in melanoma by potentiating P53 activity, and has been shown to reduce invasive activity by modulating the function of N-cadherin and focal adhesion kinase (encoded by *FAK*) [25,26]. Consistent with the involvement of GSK-3 in several signaling pathways, β-catenin knockdown only partially rescued the effect of GSK-3 inhibition on TRAIL-dependent apoptosis. Interestingly, previous studies in HeLa cells have shown that inhibition of GSK3A, but not GSK3B, enhanced sensitivity to TRAIL-mediated apoptosis [27], raising the possibility that some of the effects of CHIR99021 in our melanoma cells may be mediated through GSK3A. In pancreatic cancer, the GSK-3 inhibitor AR-18 similarly enhanced the sensitivity of pancreatic cancer cell lines to TRAIL [28]. Our findings along with previous studies implicating GSK-3 as an important regulator of TRAIL sensitivity further support the exploration of GSK-3 inhibitors as potential enhancers of the therapeutic response to TRAIL in patients.

The degree by which WNT3A enhances sensitivity to TRAIL is heterogeneous among different melanoma cell lines. The cell lines tested were heterogeneous in respect to known oncogenic mutations, with a large proportion of lines carrying the *BRAF* ^*V600E*^ mutation which is frequently found in patient tumors [29]. No specific mutations, however, were clearly associated with WNT mediated enhancement of apoptosis. While the exact determinants of apoptosis following Wnt stimulation have not been identified, regulation of the intracellular Wnt/β-catenin antagonist AXIN1 appears to play a major role in sensitivity to apoptosis. AXIN1 is a scaffold protein that is a component of the β-catenin destruction complex and modeling studies suggest that AXIN1 abundance represents the rate-limiting component of β-catenin degradation [24]. Unexpectedly, the depletion of AXIN1 significantly sensitized all melanoma cell lines to TRAIL, even those in which WNT3A was insufficient to sensitize cells to rhTRAIL. AXIN1 levels are tightly regulated by ubiquitin-mediated proteasomal degradation. Several post-translational modifications have been identified which directly affect its stability including phosphorylation by GSK-3, methylation by protein arginine methyltransferase 1 (encoded by *PRMT1*), PARsylation by tankyrase-1/2 (encoded by TNKS1/2) and SUMOylation [30–33]. Our results suggest that targeted manipulation of these pathways in such a way as to decrease AXIN1 stability could also theoretically sensitize melanoma cells to TRAIL-based therapies.

This study is the first to identify Wnt/β-catenin signaling as a regulator of TRAIL response in melanoma and supports the idea that therapeutic enhancement of Wnt/β-catenin signaling in combination with TRAIL deserves further study. Interestingly, Wnt/β-catenin signaling may provide a means to enhance TRAIL sensitivity across a broad spectrum of cancers, including diseases such as pancreatic and colorectal cancer in which activation of Wnt/β-catenin signaling has been associated with disease progression. Constitutive activation of Wnt/β-catenin signaling due to mutations in adenomatous polyposis coli (encoded by *APC*) sensitizes colonic polyps to TRAIL activation in a murine model through downregulation of cFLIP, a potent inhibitor of the extrinsic apoptotic pathway [34,35]. By contrast, WNT3A induces pro-apoptotic changes in the levels of several regulators of the intrinsic apoptotic pathway, namely BCL2L11, BBC3, and MCL1. These genes have been identified previously in both melanoma and other cells as regulators of TRAIL sensitivity [36–38]. Ultimately a better understanding of the regulation and biologic effects of Wnt/β-catenin signaling in melanoma, particularly with regards to regulating apoptosis in concert with other pathways, will allow us to better optimize therapeutic strategies in the future.

## Materials and Methods

### Reagents

Recombinant human TRAIL was purchased from Peprotech (Rocky Hill, NJ). rhTRAIL was reconstituted in PBS + 0.1% BSA per manufacturer’s instructions at 0.5mg/mL. Transient transfection of siRNA was performed with RNAiMAX, as directed by the manufacturer (13778-075, Invitrogen). siRNA sequences have been previously described [18]. Protease (#11873580001) and phosphatase (#04906845001) inhibitor tablets were purchased from Roche (Indianapolis, IN). Z-VAD-FMK was purchased from R&D systems (Minneapolis, MN cat. # FMKSP01).

### Cell Lines and Culture

The human melanoma cell lines A375, A2058, and MEL624 [18] were gifts from Cassian Yee (Fred Hutchinson Cancer Research Center, Seattle, WA). The human melanoma cell lines, COLO829, SKMEL28, and SKMEL5 were purchased from ATCC (Manassas, VA). A375 and A2058 were cultured in DMEM supplemented with 5% FBS and 1% Pen/Strep antibiotic. Melanoma lines COLO829 and MEL624 were cultured in RPMI supplemented with 10% FBS and 1% Pen/Strep antibiotic.

### RNA purification and qRT-PCR analysis

Cells were seeded in a 12 well dish. Prior to treatment with rhTRAIL and conditioned media, cells were treated with zVAD-FMK (100µM). After 24 hours, RNA was purified using the GeneJet RNA purification kit following the manufacturer’s protocol. cDNA was synthesized using RevertAid^™^ M-MuLV Reverse Transcriptase (Fermentas, Ontario, CAN). PCR was performed using a microplate-based protocol as previously described [39]

### Immunoblotting

Cells were lysed in radioimmunoprecipitation buffer (RIPA: 25 mm Tris-HCl, pH 8.0, 150 mm NaCl, 10% glycerol, 1% Triton X-100, 0.25% deoxycholic acid, 2 mm EDTA) containing protease inhibitor and phosphatase inhibitor tablets (Roche, Indianapolis, IN) Cell lysates were cleared by centrifugation and protein concentration determined by BCA assay (Pierce). 15µg of total protein was loaded per lane. Anti-cleaved CASP3 (#9661S), anti-cleaved PARP1 (#9541), anti-BCL2L11 (#2933), anti-BBC3 (#4976), anti-MCL1(D35A5), anti-Bcl-2(50E3), anti-Bcl-xL(54H6) antibodies were purchased from Cell Signaling (Cell Signaling, Beverly MA). Anti-β-tubulin (T7816) and anti-β-catenin (C2206) antibodies were purchased from Sigma Aldrich (Sigma Aldrich, St. Louis, MO). The anti-AXIN1 (AF3287) antibody was purchased from R&D Systems (Minneapolis, MN). Quantification of Western Blots was done with ImageJ Software (http://rsbweb.nih.gov/ij/index.html).

### Annexin V Apoptosis Assay

Cells were seeded in a 12 well dish and treated with the indicated conditions for 24 hours. Supernatants and trypsinized cells were collected. Cells were washed and stained with Annexin V PE and 7AAD using the Apoptosis Detection Kit I (BD Pharmigen) For experiments involving siRNAs, cells were transfected with siRNAs at a final concentration of 20nM with RNAiMax according to manufacturer’s protocol. 48 hours following transfection, cells were treated with the indicated conditions. Flow cytometry was performed using a BD FACSCanto II, and data analyzed with FlowJo 8.8.6 (Tree Star Inc, Ashland, OR) software. Experiments were performed with biological triplicates and data are representative of at least three independent experiments.

### Statistical Analysis

All experiments were performed three times, with at least three replicates per experiment. Statistical analysis was performed using GraphPad Prism software v 5.0 (GraphPad Inc., La Jolla, CA). A p-value of <0.05 was considered statistically significant.

## Supporting Information

Figure S1 **Dose response and time course melanoma cells treated with WNT3A conditioned media and rhTRAIL**.
A) A375 melanoma cells were treated with indicated doses of L or WNT3 conditioned media. The percentage of apoptotic cells was determined by Annexin V binding using FACS. Data represents the mean percentage of apoptotic cells at 24 hours post-treatment +/- SEM. B) A375 melanoma cells received no treatment or treatment with L CM or WNT3A CM (10%) + rhTRAIL (20ng/mL). The percentage of apoptotic cells was determined by Annexin V binding using FACS at indicated time points post-treatment. Data represents the mean percentage of apoptotic cells +/- SEM.
(TIF)

Figure S2 **qRT-PCR of apoptosis regulatory genes in A375 cells treated with WNT3A +/- TRAIL.**
No consistent significant differences were seen in the expression of a panel of known apoptosis-associated genes in A375 cells treated with combinations of WNT3A and rhTRAIL (20 ng/mL).
(TIF)
