# Supplementary figures and images for: Activation of Wnt/β-Catenin Signaling Increases Apoptosis in Melanoma Cells Treated with Trail
Source: PLoS One. 2013 Jul 15;8(7):e69593. doi: 10.1371/journal.pone.0069593 (PMC3711908; doi:10.1371/journal.pone.0069593)

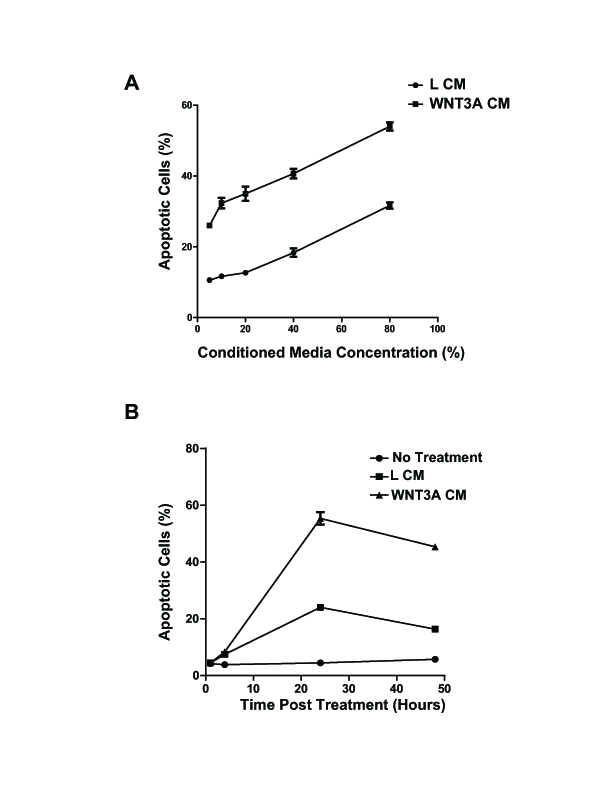

Supplement: Figure S1 — Dose response and time course melanoma cells treated with WNT3A conditioned media and rhTRAIL. A) A375 melanoma cells were treated with indicated doses of L or WNT3 conditioned media. The percentage of apoptotic cells was determined by Annexin V binding using FACS. Data represents the mean percentage of apoptotic cells at 24 hours post-treatment +/- SEM. B) A375 melanoma cells received no treatment or treatment with L CM or WNT3A CM (10%) + rhTRAIL (20ng/mL). The percentage of apoptotic cells was determined by Annexin V binding using FACS at indicated time points post-treatment. Data represents the mean percentage of apoptotic cells +/- SEM. (TIF) [file pone.0069593.s001.tif]

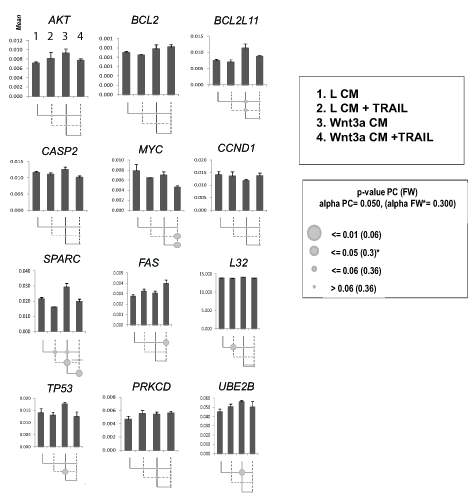

Supplement: Figure S2 — qRT-PCR of apoptosis regulatory genes in A375 cells treated with WNT3A +/- TRAIL. No consistent significant differences were seen in the expression of a panel of known apoptosis-associated genes in A375 cells treated with combinations of WNT3A and rhTRAIL (20 ng/mL). (TIF) [file pone.0069593.s002.tif]
